# GPi Oscillatory Activity Differentiates Tics from the Resting State, Voluntary Movements, and the Unmedicated Parkinsonian State

**DOI:** 10.3389/fnins.2016.00436

**Published:** 2016-09-28

**Authors:** Joohi Jimenez-Shahed, Ilknur Telkes, Ashwin Viswanathan, Nuri F. Ince

**Affiliations:** ^1^Parkinson's Disease Center and Movement Disorders Clinic, Department of Neurology, Baylor College of MedicineHouston, TX, USA; ^2^Department of Biomedical Engineering, University of HoustonHouston, TX, USA; ^3^Department of Neurosurgery, Baylor College of MedicineHouston, TX, USA

**Keywords:** Tourette syndrome, Parkinson's disease, voluntary movements, tics, globus pallidus interna, local field potentials, deep brain stimulation

## Abstract

**Background:** Deep brain stimulation (DBS) is an emerging treatment strategy for severe, medication-refractory Tourette syndrome (TS). Thalamic (Cm-Pf) and pallidal (including globus pallidus interna, GPi) targets have been the most investigated. While the neurophysiological correlates of Parkinson's disease (PD) in the GPi and subthalamic nucleus (STN) are increasingly recognized, these patterns are not well characterized in other disease states. Recent findings indicate that the cross-frequency coupling (CFC) between beta band and high frequency oscillations (HFOs) within the STN in PD patients is pathologic.

**Methods:** We recorded intraoperative local field potentials (LFPs) from the postero-ventrolateral GPi in three adult patients with TS at rest, during voluntary movements, and during tic activity and compared them to the intraoperative GPi-LFP activity recorded from four unmedicated PD patients at rest.

**Results:** In all PD patients, we noted excessive beta band activity (13–30 Hz) at rest which consistently modulated the amplitude of the co-existent HFOs observed between 200 and 400 Hz, indicating the presence of beta-HFO CFC. In all 3TS patients at rest, we observed theta band activity (4–7 Hz) and HFOs. Two patients had beta band activity, though at lower power than theta oscillations. Tic activity was associated with increased high frequency (200–400 Hz) and gamma band (35–200 Hz) activity. There was no beta-HFO CFC in TS patients at rest. However, CFC between the phase of 5–10 Hz band activity and the amplitude of HFOs was found in two TS patients. During tics, this shifted to CFC between the phase of beta band activity and the amplitude of HFOs in all subjects.

**Conclusions:** To our knowledge this is the first study that shows that beta-HFO CFC exists in the GPi of TS patients during tics and at rest in PD patients, and suggests that this pattern might be specific to pathologic/involuntary movements. Furthermore, our findings suggest that during tics, resting state 5–10 Hz-HFO CFC shifts to beta-HFO CFC which can be used to trigger stimulation in a closed loop system when tics are present.

## Introduction

Tourette syndrome (TS) is a neuropsychiatric disorder defined by the presence of vocal and motor tics, but characterized by frequent co-morbidities such as attention deficit disorder and obsessive compulsive disorder (Jankovic, 2001). Onset is typically in childhood with a waxing and waning course that is likely to resolve or significantly improve by the late teenage years (Freeman and Tourette Syndrome International Database, 2007). While the worst-ever time period for tics is often 10–12 years of age (Bloch et al., 2006a; Shprecher et al., 2014), 5–10% of patients will continue to experience significant or worsening symptoms into adulthood (Freeman and Tourette Syndrome International Database, 2007).

Predictors of severity or the course of TS are not well understood, though contributing factors may include presence of fine motor skills deficits (Bloch et al., 2006b), reduced caudate volumes (Bloch et al., 2005) or greater tic severity at a younger age (Bloch et al., 2006a). Approximately 5% of individuals with TS in a tertiary referral setting may meet criteria for “malignant” TS in which symptoms are self-injurious or may lead to emergency room visits or hospitalizations (Cheung et al., 2007). Pharmacologic and non-pharmacologic therapies exist for management of TS symptoms, and several new treatments are currently being investigated (Kious et al., 2016).

Deep brain stimulation (DBS) is an emerging therapy for advanced, medication-refractory TS, and consensus criteria for DBS candidacy have recently been revised (Schrock et al., 2015). Considerations include such factors as age, tic severity, tics as the primary source of disability, failure of typical medications and behavioral therapy, psychiatric co-morbidities, and psycho-social factors. While the exact mechanism of action of DBS is unclear, small series have suggested that marked improvement can be achieved (Schrock et al., 2015). Randomized clinical trials (Maciunas et al., 2007; Ackermans et al., 2011; Kefalopoulou et al., 2015) have shown less robust improvements following DBS, but this could be related to difficulties in designing clinical trials to adequately study this complex condition (Jimenez-Shahed, 2015). As a consequence, no consensus exists regarding the optimal site of stimulation in TS. The most studied targets include the globus pallidus interna (GPi) and the centromedian-parafascicular complex of the thalamus (Cm-Pf).

During DBS, an electrical stimulus is applied to a deep nucleus relevant to movement generation and the pathogenesis of tic activity. As with DBS in other movement disorders, a recording microelectrode is advanced into the target while electrical recordings (microelectrode recordings, or MER) are obtained, which are used to identify the optimal location for final electrode placement. MER allows for analysis of single unit neuronal activity (SUA), and different nuclei can be identified by their signature firing rate and pattern, which may also differ by disease state (Gross et al., 2006). Concurrent to the recording and analysis of SUA (Telkes et al., 2015), and also following placement of the DBS macroelectrode (Telkes et al., 2014), local field potential (LFP) recordings can be obtained. LFPs represent the aggregate activity of a group of neurons within the target structure. LFP recordings can also be obtained from the implanted DBS electrode(s) during surgery for replacement of a depleted implantable pulse generator (IPG; Abosch et al., 2012).

LFP analysis in Parkinson's disease (PD) has provided substantial insight into the pathophysiology and treatment of this movement disorder, including identification of the beta band of oscillations as a potential biomarker for the untreated disease state, which is abolished after administration of levodopa (Thompson et al., 2014). Similarly, analysis of intraoperative LFP recordings obtained during DBS electrode placement offers a unique opportunity to study the *in vivo* neurophysiology of tics in TS.

The purpose of this study was to obtain LFP recordings during the resting state, during tic activity, and during voluntary movements in patients with TS, and to compare these recordings to those obtained in the unmedicated resting state of patients with PD undergoing the same surgical procedure in the same brain target. The chosen site of stimulation for TS at our center is the bilateral postero-ventrolateral portion of the globus pallidus interna (pvGPi), based upon our prior experiences (Shahed et al., 2007) and anatomic considerations relating this nucleus to basal ganglia circuitry and TS phenomenology (Viswanathan et al., 2012), including “dystonic” tics. For a number of years, pvGPi DBS has also been a well-established treatment for patients with advanced PD (Deep-Brain Stimulation for Parkinson's Disease Study Group, 2001; Weaver et al., 2009). We hypothesized that LFP spectral characteristics and non-linear interactions between different frequency bands can distinguish tic activity from the resting and parkinsonian states, as well as from voluntary movements.

## Methods

Patients with medication refractory TS are considered for surgery at our center during consensus review by a team of Movement Disorders Neurologists, Neurosurgeons, and Neuropsychologists, and according to accepted criteria. In both TS and PD patients, comprehensive pre-operative neurologic and neuropsychological assessments are performed and reviewed by the consensus team in order to determine the appropriateness of DBS for each candidate. Experimental procedures related to the recording and analyses of LFPs are approved by the Institutional Review Board of Baylor College of Medicine. Candidates for DBS confirmed by consensus review were approached for participation in the study and written informed consent was obtained.

For both TS and PD patients undergoing an electrode placement procedure, the stereotactic coordinates for the pvGPi are chosen based on direct targeting (Machado et al., 2006). The microelectrode is advanced to the intended target using standard MER techniques. After the optimal location for implantation of the electrode is decided, the DBS macroelectrode (model 3387, Medtronic, Minneapolis, MN) is inserted to the appropriate depth. In PD patients, the electrode placement procedure is performed while “off” medications.

LFP recordings are obtained during the DBS electrode placement procedure in the hemisphere contralateral to the more severe tic activity in TS subjects, and in the left hemisphere of all PD subjects. The DBS macroelectrode is connected to sterile recording cables [a twist-lock cable (Medtronic) and a custom design matching cable (BioCables)] and the LFP recordings are obtained from its four contacts by using a gHIAmp (gTec, Graz, Austria) biosignal amplifier at a sampling rate of 1200 Hz, with 24-bit A/D resolution. Electromyography and electrocardiography is also performed in order to monitor the patient's behavioral state and to identify and remove artifacts from recordings. These signals are entered into the multipurpose neural data acquisition system in order to synchronize behavior with the neural data. For TS patients, video recordings are also made to characterize the phenomenology of any involuntary movements and synchronize LFP activity to the presence, absence, onset and offset of movements. During an IPG replacement procedure, the depleted IPG is first disconnected from the DBS lead extension cables. The distal ends of the extension cables are then connected to sterile recording cables (multiplex adapter cable model 74001 [2x4] and a twist-lock cable (model 3550-03; Medtronic). LFP recordings are obtained thereafter using the same biosignal amplifier, accompanied by electromyography, electrocardiography, and video recordings.

LFPs are recorded before intraoperative test stimulation during a resting period lasting for a minimum of 1 min in both TS and PD patients, and for TS patients, during provocation of spontaneous tics and during voluntary movements. Tic provocation is accomplished by suggestion (discussing tic characteristics or other triggers to provoke spontaneous tic activity). At least 3 repetitions of this sequence are performed. The recordings and motor assessments are conducted over a 10–15 min period. A representative video demonstrating the data and video acquisition system is included in the Supplementary Material (Video 1). Once LFP recordings are complete, the recording cable is disconnected and usual surgical procedures continue, including intraoperative testing of stimulation (Machado et al., 2006), to verify the presence of motor benefits and absence of stimulation side effects.

In this investigation, we report LFP analysis from three adult patients with TS and four with PD. LFP recordings from one TS subject (Subject III) were made during an IPG replacement procedure, 3.0 years after initial DBS electrode placement surgery. All other recordings were made during the initial DBS electrode placement surgery.

## Signal processing

All recorded signals were visualized with custom software that was developed in-house, and annotated to distinguish artifact and/or epochs of resting, active movements, and tic activity. Based on these annotations, resting state data from all subjects and tic periods from TS subjects were extracted into MATLAB (Mathworks, Natick, Massachusetts). LFP data from all four contacts were band-pass filtered using an FIR filter with 3 and 500 Hz cutoff frequencies. For elimination of power line artifacts, a 60 Hz notch filter and notch filters at harmonics of 60 Hz were used. During preprocessing of LFP data, raw signals were converted into a bipolar derivation (contacts 0–1, 1–2, 2–3).

The frequency content of the oscillatory LFP activity from the GPi was explored by power spectral analysis. The power spectrum of each bipolar LFP derivation was estimated using the modified Welch periodogram method (Telkes et al., 2014). Specifically, a fast Fourier transform (FFT) was computed with a 2048-sample long Hanning window and the window was shifted with 50% overlap. Since there were multiple segments of tic movements, the power spectra related to each state were averaged. In order to compare the power changes in specific LFP sub-bands between different events, the power in the beta, gamma and high frequency oscillation (HFO) ranges were computed over averaged tic and averaged voluntary hand movement periods separately, and were normalized according to the power of the baseline. The power changes with respect to baseline were represented in decibel (dB) scale,

(1) $$\begin{array}{r} P_{i,j,k}\left(dB\right)=10log_{10}\left(\frac{A_{k}}{R_{k}}\right) \end{array}$$

where *A*_*k*_ and *R*_*k*_ represent the active and resting state power of sub-band indexed with *k* respectively.

In order to quantify the non-linear interactions between different LFP frequency bands, the coupling between the amplitude of HFOs and the phase of low frequency oscillations were investigated by using a phase locking value (PLV) approach (Lachaux et al., 1999). For this particular purpose, LFP signals were filtered with a 2nd order Butterworth filter from 4 to 40 Hz with a 2 Hz band width and 1 Hz shift that constituted 37 bandpass filtered components for the low frequencies. Similarly, the same LFP signal was bandpass filtered from 150 to 500 Hz with another 2nd order Butterworth filter with 80 Hz band width and 25 Hz shift. Thus, 15 bandpass filtered components were obtained for high frequencies. The envelope of these high frequency components was extracted by using the Hilbert transform and the PLV method was used to estimate cross-frequency coupling (CFC) between the phase of low frequency activity and the amplitude of high frequency activity.

An analysis for statistical significance was performed over every single CFC calculated in order to check if the observed value differed from what would be expected due to chance alone. To achieve this, a surrogate analysis was performed by calculating the coupling between randomly selected blocks of both amplitude and phase envelopes. The chance occurrence of coupling between phase and amplitude was estimated by using 100 surrogates, and a z-score was computed for each individual CFC. In order to account for multiple comparisons, Bonferroni's correction was applied (the significance level of the test α = 0.05/555, where the number of tests = 37 × 15, or 555).

## Results

The clinical characteristics of enrolled subjects are included in Table 1. Based upon the tic characteristics and the LFP recording environment (electrode placement surgery vs. IPG replacement), handgrip movements were performed in two subjects and lateral neck movements were used in the other, in order to investigate voluntary movements. The type of voluntary movements and observed tic epochs and their duration are provided in Table 2. In all subjects where recordings were made during DBS surgery, the position of the electrode at the time of LFP recording remained unchanged after intraoperative testing of stimulation verified improvement in symptoms and absence of side effects. Test stimulation was performed after LFP recordings were made. Additionally, a contralateral DBS electrode was also successfully placed, and post-operative programming of the device led to reduction in motor symptoms (Table 1).

**Table 1:** **Clinical Characteristics of Subjects receiving pvGPi stimulation**.

	**Diagnosis**	**Age at time of surgery, gender**	**Pre-op motor exam**	**Motor exam 1-year post-implant (% change)**	**Hemisphere**
Subject I	TS	36 yo M	YGTSS total: 84	YGTSS total: 67 (20.2%)	Left
Subject II	TS	27 yo M	YGTSS total: 88	YGTSS total: 58 (34.1%)	Left
Subject III	TS	22 yo F	YGTSS total: 81	YGTSS total: 45 (44.4%)	Left
Subject IV	PD	51 yo M	MDS-UPDRS3: Off = 72; on = 18	–	Left
Subject V	PD	49 yo F	MDS-UPDRS3: Off = 47; on = 28	–	Left
Subject VI	PD	62 yo M	UPDRS3: Off = not done; on = 40	–	Left
Subject VII	PD	62 yo M	MDS-UPDRS3: Off = 40; on = 19	–	Left

TS, Tourette syndrome; PD, Parkinson's disease; YGTSS, Yale Global Tic Severity Scale; MDS-UPDRS3, Movement Disorders Society Unified Parkinson's Disease Rating Scale Part 3 Motor exam; UPDRS, Unified Parkinson's Disease Rating Scale.

**Table 2:** **Data segments used for the estimation of power spectra and CFC results in TS subjects**.

	**Tic activity**		**Voluntary hand movements**	
	**No. of epochs**	**Total duration (s)**	**No. and type of movements**	**Total duration (s)**
Subject I	3	35-17-10	3 sets of handgrips (5 times each)	11-9-8
Subject II	5	25-36-17-27-18	3 sets of handgrips (10, 5, 5 times)	18-11-15
Subject III	2	193-122	2 sets of lateral neck movements (4 and 6 times)	16-15

**Figure 1:**
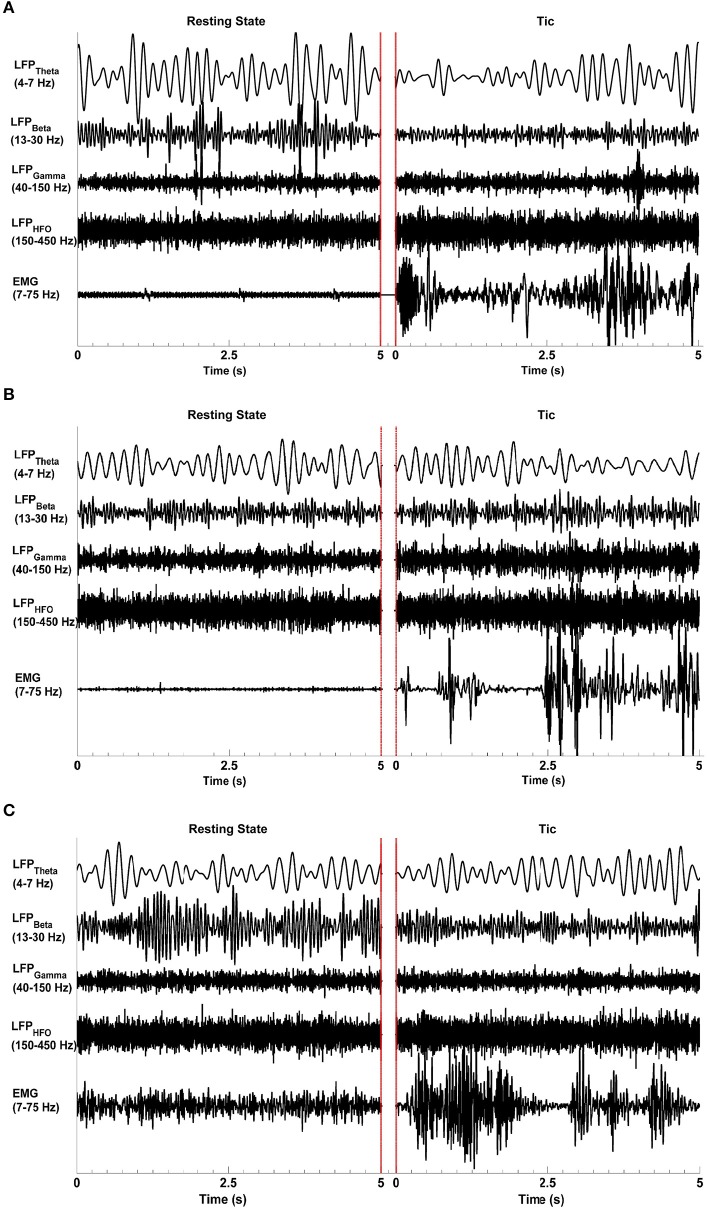
**Representative LFP signal characteristics during the resting state and tics**. Epochs of each state lasting 5 s are presented, separated by red lines. The raw LFP data was bandpass filtered at the theta (4–7 Hz), beta (13–30 Hz), gamma (40–150 Hz), and high frequency oscillation (HFO, 150–500 Hz) ranges. The EMG signals were filtered between 7 and 75 Hz. Event-related desynchronization and synchronization are evident in Subjects I **(A)** and III **(C)** but not in Subject II **(B)**. All subjects demonstrate a clear increase in EMG activity during tics compared to the resting state. The red lines indicate the end of one epoch and the beginning of another.

Figure 1 shows representative raw LFP signal characteristics in all three TS patients (Subjects I–III) in the resting state (left-hand side) when no tics or other movements were present, and during tic activity (right-hand side) as characterized by video and surface EMG. The beta, gamma, and high frequency bands are associated with event related desynchronization (ERD) and synchronization (ERS) during tic events. The LFP data filtered between 13 and 30 Hz in Subject I and Subject III indicate the presence of ERD, with lower amplitude beta band oscillations during tic periods compared to the resting state. During tic periods, there is also amplitude enhancement (ERD) in the gamma range (40–150 Hz) and higher frequencies (150–500 Hz) in all subjects. LFP raw data from Subject II filtered in the same frequency bands do not show an ERD-ERS pattern. A clear difference in theta range (4–7 Hz) oscillations between resting and tic periods are also not apparent in Subject II, whereas EMG signals show a clear difference between resting and tic states in all subjects.

**Figure 2:**
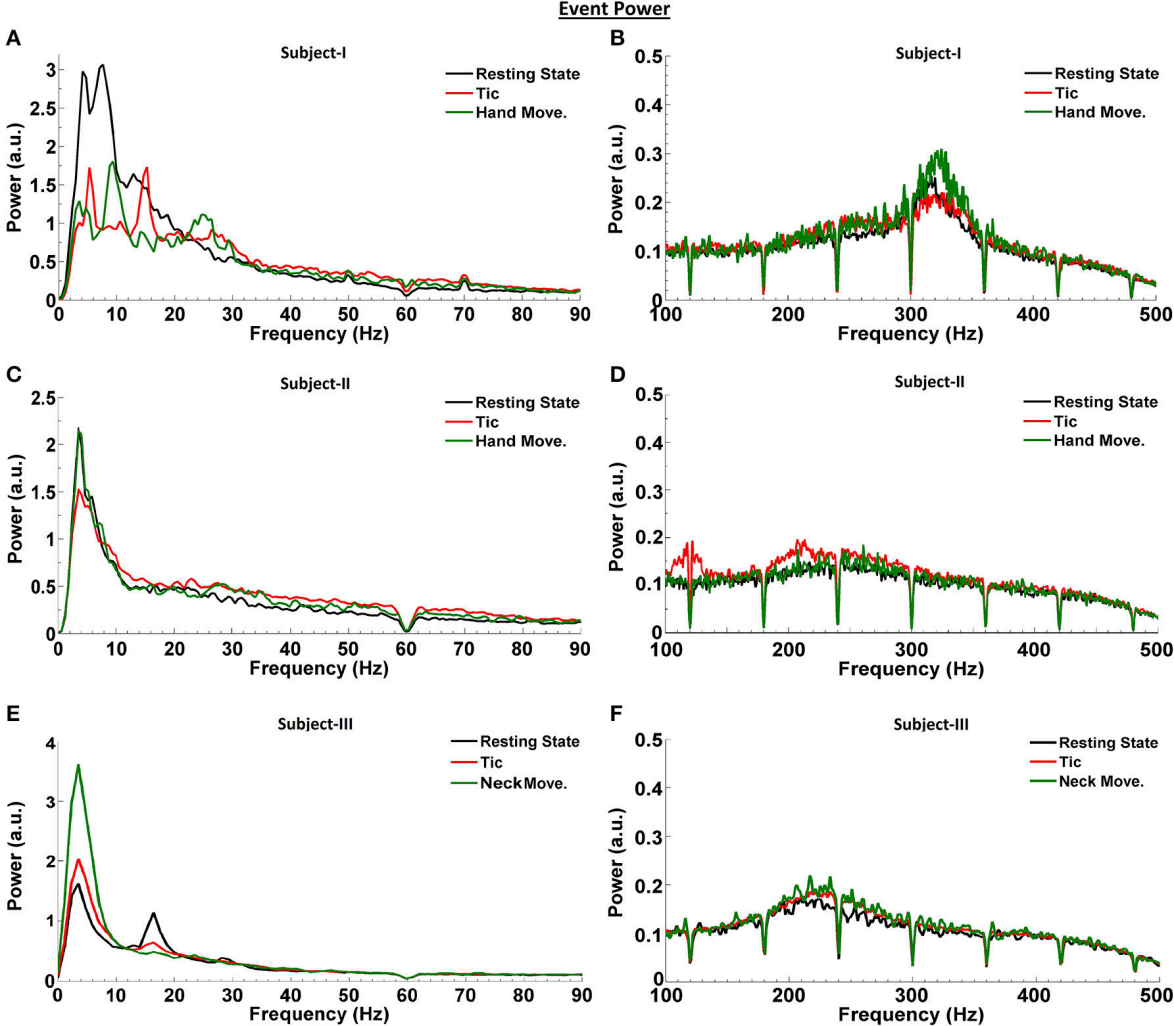
**LFP power spectra at rest, during tic activity and during voluntary movements in TS subjects**. Spectra are shown in the bipolar contact derivation with the highest power [0–1 (Subjects I and II) and 1–2 (Subject III)]. LFP activity related to the resting state is shown in black, related to tic activity is shown in red, and related to voluntary movements is shown in green, for Subject I **(A,B)**, Subject II **(C,D)**, and Subject III **(E,F)**. The number and the duration of individual tic epochs and active movements which were averaged for spectral analysis are provided in Table 2.

We first investigated the LFP power spectra from each bipolar electrode combination (0–1, 1–2, and 2–3) in Subjects I–III, at rest and during tic activity. The greatest power of LFP spectral dynamics was most commonly found in 0–1 and 1–2 bipolar contact derivations. Figure 2 compares the power spectra in the resting state to that during tic activity and active movement, obtained from the bipolar contact combination with the highest LFP power and across frequency ranges. In Subject I (Figures 2A,B), 4 Hz (theta band) and 13 Hz activity (beta band) during rest switches to 5 and 15 Hz activity during tics, and shifts further during voluntary hand movements to a clear peak at 10 Hz with enhancement in the beta band between 20 and 30 Hz. LFP power is broadly decreased in the 13–30 Hz range during voluntary hand movements, correlating with the ERD pattern demonstrated in Figure 1. In the HFO range, voluntary hand movements have the highest power (fast HFO) compared to the resting state and tic events. LFP power during tics is broadly higher than baseline between 150 to 450 Hz.

In Subject II, a 3.5 Hz activity peak is consistently seen during all events (Figures 2C,D). Even though gamma-ERS occurs during voluntary hand movements, no clear peak is observed. The power of gamma LFPs during averaged tic periods is higher than during the resting state. There is also clear enhancement of slow HFO activity during tic events compared to both baseline and voluntary hand movements. There are no event-related differences in the fast HFO range. In Subject III, compared to the resting state, the power of the theta peak is the highest during active movements, and is only slightly higher during averaged tic periods (Figures 2E,F). A clear beta peak is observed during rest, which attenuates during averaged tic periods and active movement. Once again, the power in the HFO range is higher during tics and active movement compared to the resting state.

**Figure 3:**
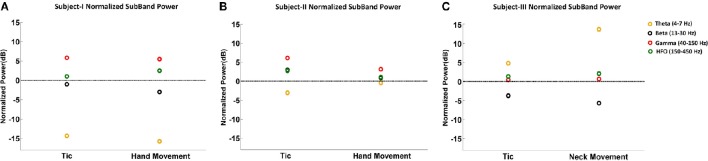
**Normalized sub-band power of LFPs during tic and voluntary hand movements relative to the resting state**. The sub-band power changes at theta (4–7 Hz), beta (13–30 Hz), gamma (40–150 Hz), and HFO (150–450 Hz) bands are shown for Subject I **(A)**, Subject II **(B)**, and Subject III **(C)**.

Figure 3 summarizes the LFP power changes during tics and voluntary hand movements across frequency sub-bands relative to the resting state in each of three TS subjects. Theta band changes relative to the resting state are inconsistent across subjects. Beta band activity was lower during tics and lowest during voluntary movements in Subjects I and III, corresponding to the ERD demonstrated in Figure 1. By contrast, neither reduced beta activity nor gamma-ERS during movement events were seen in Subject II. HFO activity was consistently higher during tics in all subjects. In Subjects I and III, there is a further increase in power of this frequency sub-band during voluntary movements.

**Figure 4:**
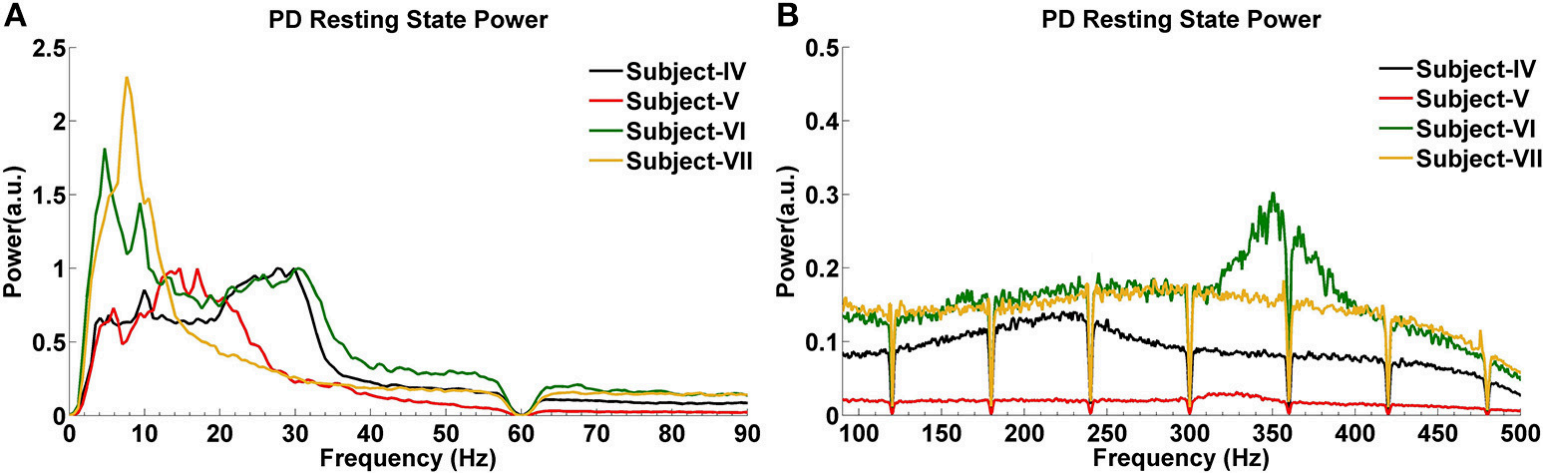
**LFP power spectra of PD subjects in the resting state at low (A) and high (B) frequencies**.

The GPi power spectrum in the resting state for four subjects with PD is shown in Figure 4. Two PD Subjects (V and VI) show increased activity around 5 Hz, and one (Subject VII) has a low power peak at 7 Hz. In the beta band, Subjects IV and VI have increased activity between 20 and 30 Hz, while Subject V demonstrates a relatively large and wide range of beta band activity across the 12–30 Hz spectrum. Subject VII shows weak beta band activity. In the HFO range, all PD subjects show broad increases, with two (IV and VII) in the slower HFO spectrum (200–250 Hz) and two (V and VI) in the faster spectrum (250–350 Hz).

**Figure 5:**
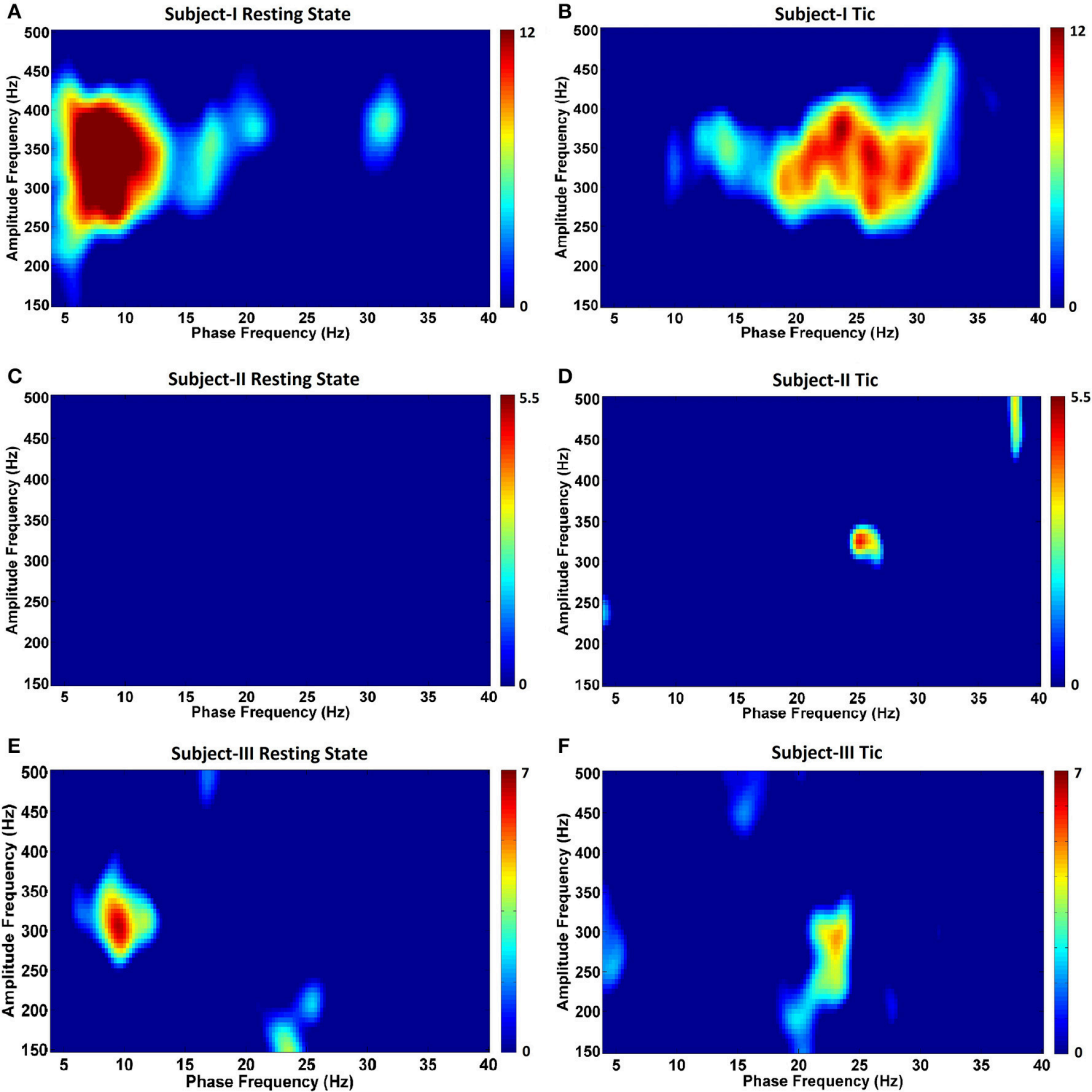
**Phase-amplitude coupling comodulograms for three TS subjects during the resting state and tic activity**. Panels **(A–F)** demonstrate comodulograms for Subjects I–III respectively.

**Figure 6:**
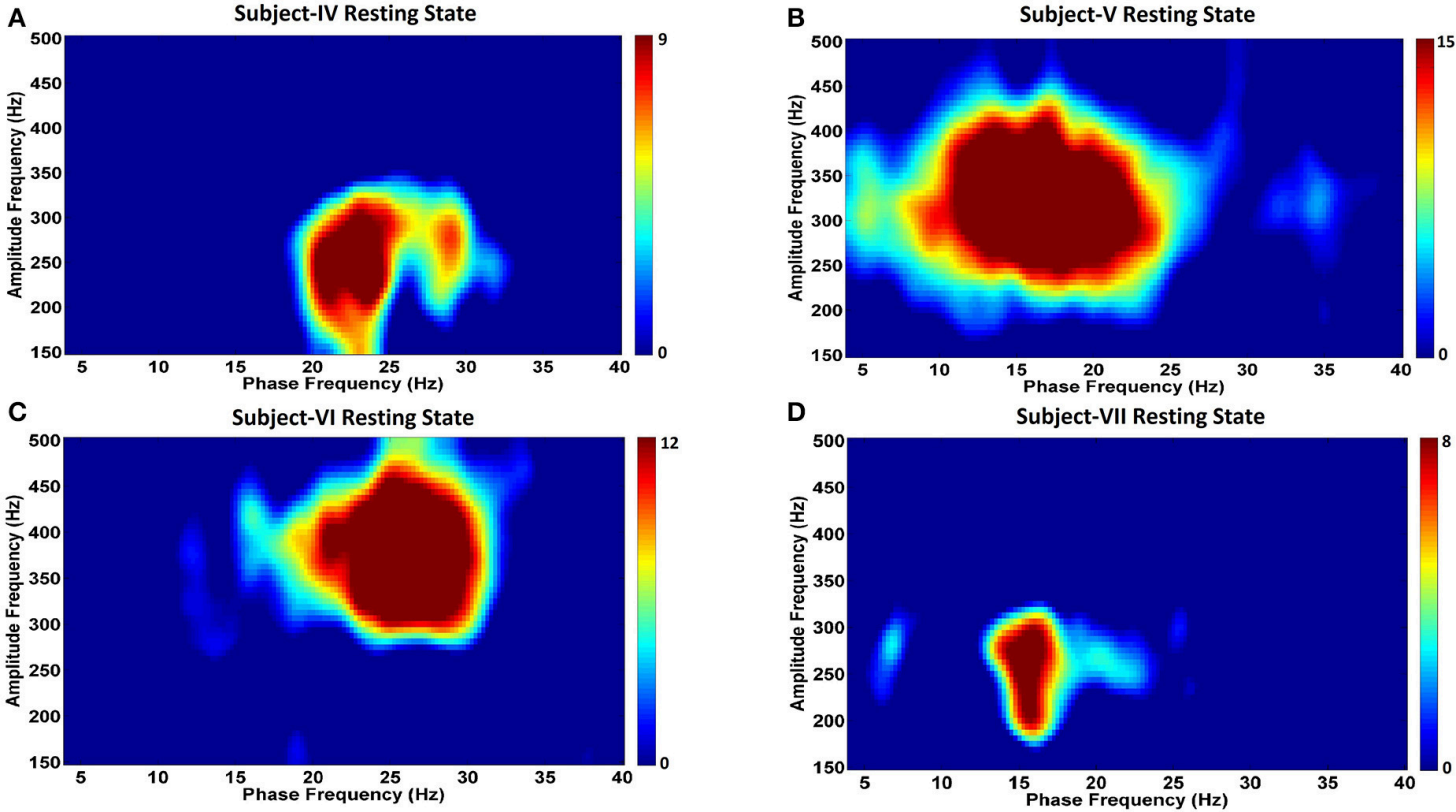
**Phase-amplitude coupling comodulgrams for four PD subjects in the resting state**. Panels **(A–D)** demonstrate the resting state comodulograms in Subjects IV–VII, respectively, who all have Parkinson's disease, and are “off” medications.

Next, in order to characterize the temporal relationships between low frequency (4–40 Hz) and wide HFO (150–500 Hz) bands, we assessed for the presence of CFC. Figure 5 depicts the GPi CFC comodulograms for each TS subject, and Figure 6 for each PD subject. In subjects with TS, CFC was investigated at rest (Figures 5A,C,E) and during tics (Figures 5B,D,F). The strongest CFC was seen in Subject I during the resting state, at a phase frequency between 5 and 10 Hz, coupled to the HFO amplitude at 250–400 Hz (*p* < 0.05) (Figure 5A). Interestingly, this range overlaps substantially with the theta range LFP frequency peaks seen during the resting state in this individual (Figure 2A). Significant CFC was also seen during tics, but at a higher beta frequency phase (13–33 Hz, *p* < 0.05) and remaining coupled to the amplitude in the same range of HFOs (Figure 5B). This phase coupling was maximal at 24–26 Hz.

By contrast, no CFC was found in Subject II during the resting state (Figure 5C). CFC during tic periods was weak, but still statistically significant (*p* < 0.05) (Figure 5D), and still demonstrated coupling between the phase of beta activity (localized at 25 Hz) and the amplitude of HFOs (localized at 325 Hz). In Subject III, the resting state (Figure 5E) is characterized by CFC between the phase frequencies localized at 8–10 Hz, and coupled to the amplitude of HFOs at 275–325 Hz (*p* < 350 Hz), whereas tic periods (Figure 5F) are characterized by a shift to beta-HFO CFC to the phase of beta activity localized at 23–24 Hz, and coupled to the amplitude of HFOs at 275–300 Hz (*p* < 0.05). The presence of CFC was also assessed during voluntary movements in all subjects, and none was found across any frequency range (data not shown).

Analysis of CFC in PD patients during the resting state indicates a very strong phase-to-amplitude modulation between the phase of wide beta band activity and the amplitude of HFOs (Figures 6A–D). CFC phase frequencies are consistent with the increased LFP power shown in Figure 6, as are the amplitude frequencies for HFOs. For example, Subject V (Figure 6B) shows stronger and more widespread activity from 10 to 25 Hz than the other PD subjects, which corresponds to the widest phase frequency of CFC. This is coupled to the amplitude of HFOs ranging from 250 to 400 Hz, a range where the LFP power spectra is also greatest (Figure 4). On the other hand, Subject VII had the lowest LFP spectral power without a clear beta frequency band peak. This corresponds to the smallest coupling amongst PD subjects, though it was still significant (*p* < 0.05). HFOs were also amongst the lowest frequency range (Figure 4).

## Conclusions

In this study we explored the LFP characteristics in the pvGPi of three patients with TS and four patients with PD. Although theta frequency peaks, lesser beta peaks, and HFO activity characterized the resting state in TS subjects, changes in LFP sub-bands did not consistently differentiate tics from active movements or the resting state. However, we did find a substantial difference in CFC between tic periods compared to the resting state, which was not seen during voluntary movements. Specifically, in TS subjects at rest, we found coupling between the phase of theta-low alpha oscillations to the amplitude of HFOs in 2 subjects, and during tic activity we found coupling between the phase of beta oscillations to the amplitude of HFOs in all 3 subjects. Amongst unmedicated PD patients at rest, we demonstrated increased beta band and broad HFO activity, as well as CFC between the phase of these beta oscillations and the amplitude of HFOs.

It is well-recognized that beta frequency oscillations are characteristic of the “off” medication state in PD in both the STN and GPi, and are abolished in the “on” state after administration of levodopa (Brown et al., 2001). The degree of improvement in motor symptoms (bradykinesia and rigidity) correlates with the degree of beta band suppression (Weinberger et al., 2006; Ray et al., 2008; Kuhn et al., 2009). Lastly, in the STN, beta ERD occurs upon movement initiation and heightened synchronization occurs upon termination of movements (Alegre et al., 2005; Hsu et al., 2012). As expected, we found excessive beta band activity at rest in our PD subjects with lower power HFOs across a broad range. By contrast, TS is a hyperkinetic movement disorder without features of bradykinesia or rigidity, with abnormal movements occurring in bouts. When the LFP spectra of TS subjects were compared to those of PD subjects, the resting state was found to be characterized by predominant excessive theta activity (3.5 or 4 Hz) in all three subjects, and also with HFOs (200–400 Hz) at lower power. In PD subjects, the main energy of LFPs was in the higher frequencies, except for Subject VI, who also shows high activity ranging from 5 to 13 Hz. Two TS subjects demonstrated beta frequency oscillations but at a lower power than the theta peak. Our findings therefore support the idea that beta band activity relates to relative akinesia.

Previous investigations into the LFP characteristics of TS patients undergoing DBS have focused on the thalamus. Neumann et al. (2013) identified LFP patterns in 5 subjects undergoing GPi and CM-Pf DBS, and found a peak in the 6–10 Hz frequency band. Others have also demonstrated alpha (8–13 Hz) or lower frequency (2–7 Hz) activity in the VO nucleus of the thalamus (Marceglia et al., 2010) and CM-Pf (Maling et al., 2012; Bour et al., 2015), but the clinical correlates of these frequency sub-bands remain unclear.

In PD, however, the power of the gamma and HFO spectra increases after levodopa administration, and gamma activity increases in the “off” state during voluntary movement (Brown et al., 2001; Foffani et al., 2003; Kane et al., 2009). Moreover, a cross-frequency coupling (CFC) between the phase of beta oscillations and the amplitude of HFOs has been identified in the STN, which also attenuates following administration of levodopa and is less prominent in patients with milder PD symptoms (Lopez-Azcarate et al., 2010; Ozkurt et al., 2011; Van Wijk et al., 2016). Coupling between phase of beta band oscillations and amplitude of broad band gamma activity (50–200 Hz) has also been demonstrated in the motor cortex of PD patients, but found to be absent in dystonia and epilepsy (De Hemptinne et al., 2013), and is abolished after DBS (De Hemptinne et al., 2015). Gamma band and HFOs therefore were suggested to represent a prokinetic state, while beta-HFO phase amplitude coupling were thought to characterize relative akinesia in patients with PD.

Our investigation identifies the presence of beta-HFO CFC in the pvGPi of four PD subjects during the unmedicated resting state. To our knowledge, this finding has not been previously reported. We were further able to demonstrate, for the first time, that the pattern of CFC in TS subjects at rest differs from that of unmedicated PD, and that this in turn differs from the CFC during tics. In two of three TS subjects, CFC between the phase of 5–10 Hz band activity and the amplitude of HFOs was found during the resting state. The 5–10 Hz range highly overlaps with the excessive theta band activity we observed at rest (Figure 2). We further found that this resting state CFC in the same subjects shifts to beta-HFO CFC during tics. Despite the obvious change in the phase index of CFC between the resting state and tic periods, we were unable to identify a consistent or corresponding change in theta band power during voluntary movements or tic periods, compared to the resting state (Figure 3). It is possible that the methodologic differences in LFP capture in Subject III (recorded during IPG exchange) may have contributed to this inconsistency. Nonetheless, the consistent beta-HFO CFC seen during tic periods in all three subjects is notable, though found to a lesser extent in Subject II. LFP analysis in Subject II did not show either beta-ERD or gamma-ERS relative to the resting state during movements or any CFC while at rest. Since LFPs recorded from motor territories of the basal ganglia during voluntary movements are associated with beta-ERD (Kuhn et al., 2004), the absence of beta-ERD and gamma-ERS in Subject II suggests that the electrode might not be optimally placed within the pvGPi motor territory, thereby explaining the lack of CFC at rest or strong CFC during tics.

Dystonia is another movement disorder amenable to pvGPi DBS that is characterized by diminished power in the beta band and higher power in the 8–20 Hz range (Silberstein et al., 2003; Weinberger et al., 2012). Liu et al. (2008) showed an increased power of synchronization in the 3–18 Hz range during dystonic spasms compared to the resting state. Neumann et al. (2016) demonstrated that theta (but not beta) frequency peaks in patients with cervical dystonia at rest correlated with symptom severity. CFC in dystonia patients within the GPi has been identified between the phase of theta and the amplitude of gamma oscillations (Moll et al., 2014), though it is unclear if this was found at rest or during dystonic spasms. Barow et al. (2014) further demonstrated that high frequency stimulation of the pvGPi can suppress theta oscillatory activity in subjects with mobile, phasic dystonia. Excessive theta activity in the pvGPi at rest in both dystonia and TS patients may therefore represent an underlying pathophysiologic similarity that supports the notion that a “dystonic” phenomenology of tics may indeed be treated with pvGPi DBS, similar to idiopathic dystonia, and as demonstrated by our three cases.

Another recent investigation into the LFP dynamics of the subthalamic nucleus (STN) in both PD and dystonia patients (Wang et al., 2016) found similar power spectral densities across multiple frequency ranges, including beta and theta, between the two groups, as well as resting state beta-HFO phase-amplitude coupling. Rather than representing a PD biomarker, this coupling may therefore represent pathologic network activity in patients with movement disorders (such as PD, dystonia and TS), though studies in patients without movement disorders are lacking.

Although the present study is limited to three patients with TS, and lack of information about the response of these LFP and CFC patterns to medications or DBS, our findings have particular relevance to the design of a closed loop, on-demand DBS system based on sensing of *in vivo* neurophysiologic biomarkers of involuntary movements such as tics. Further investigation to distinguish the LFP characteristics between tic and voluntary movement sequences is warranted in order to make the sensing paradigms as precise as possible. However, our findings do suggest the possibility that the shift in phase to amplitude CFC from the resting state (5–10 Hz activity coupled to HFO) to tic activity (beta-HFO) can be used to trigger stimulation in a closed loop system when tics are present. Furthermore, our work provides support for the continued investigation pvGPi DBS in cases of refractory TS as an overall modulator of tics.

## Author contributions

JJS, IT, AV, NI: Substantial contributions to the conception or design of the work; substantial contribution to the acquisition, analysis, or interpretation of data for the work; Drafting the work and revising it critically for important intellectual content; Final approval of the version to be published; Agrees to be accountable for all aspects of the work in ensuring that questions related to the accuracy or integrity of any part of the work are appropriately investigated and resolved.

### Conflict of interest statement

JJS has received consulting fess from Medtronic, Inc., and St. Jude Medical. The other authors declare that the research was conducted in the absence of any commercial or financial relationships that could be construed as a potential conflict of interest.

## Supplementary material

Video 1 **This video segment demonstrates a representative example of a tic flurry recording during DBS electrode placement into the pvGPi of Subject II**. An initial resting period is followed by the onset of a series of repetitive right hand tics. The real-time neural acquisition system captures raw LFP signals in each bipolar electrode derivation, EMG activity, and an electrocardiogram.
